# Subcutaneous Panniculitis-Like T-cell Lymphoma With Scalp Involvement: A Diagnostic Challenge With Lupus Panniculitis

**DOI:** 10.7759/cureus.110523

**Published:** 2026-06-09

**Authors:** Younes Tamim, Yassine Berrada, Farah El Hadadi, Kaoutar Znati, Mariame Meziane, Nadia Ismaili, Benzekri Laila, Karima Senouci

**Affiliations:** 1 Dermatology, Ibn Sina University Hospital Center/Mohammed V University, Rabat, MAR; 2 Pathology, Ibn Sina University Hospital Center/Mohammed V University, Rabat, MAR

**Keywords:** alopecia, cutaneous t-cell lymphoma, immunohistochemistry, lupus panniculitis, scalp involvement, subcutaneous panniculitis-like t-cell lymphoma

## Abstract

Subcutaneous panniculitis-like T-cell lymphoma (SPTCL) is a rare primary cutaneous cytotoxic T-cell lymphoma that may show marked clinical and histopathological overlap with lupus panniculitis. We report the case of a 22-year-old woman with a three-year history of slowly progressive depressed hyperpigmented lipoatrophic plaques involving the thighs, back, and left upper limb, associated with an erythematous alopecic scalp plaque. An initial thigh biopsy showed interface dermatitis, dermal mucin deposition, and lobular lymphocytic panniculitis with positive direct immunofluorescence, leading to a diagnosis of lupus panniculitis. Immunological investigations showed positive antinuclear antibodies and anti-double-stranded DNA antibodies. A second thigh biopsy demonstrated lobular panniculitis with atypical lymphoid cells rimming adipocytes. Immunohistochemistry showed CD3+, CD8+, CD4-, and CD56- cells with positive perforin and granzyme B expression and a Ki-67 index of 40%, consistent with SPTCL. Re-evaluation of the first biopsy supported the same diagnosis. Subsequent biopsy of the progressively enlarging ulcerated scalp lesion confirmed persistent scalp involvement by SPTCL. Oral corticosteroids induced an initial response, but relapse occurred one year later, and methotrexate was added. This case highlights the marked diagnostic overlap between lupus panniculitis and SPTCL and the uncommon scalp localization of SPTCL.

## Introduction

Subcutaneous panniculitis-like T-cell lymphoma (SPTCL) is a rare primary cutaneous lymphoma characterized by lobular panniculitis composed of neoplastic cytotoxic CD8+ T lymphocytes [1-3]. According to the 2018 WHO-EORTC classification, SPTCL is a distinct clinicopathologic entity with a generally more favorable prognosis than primary cutaneous gamma-delta T-cell lymphoma [3]. Clinically and histologically, SPTCL may closely overlap with lupus panniculitis, creating significant diagnostic challenges [4,5]. Overlapping features, such as lobular panniculitis, interface dermatitis, dermal mucin deposition, and positive direct immunofluorescence, may initially suggest lupus panniculitis [4,5]. In addition, atypical presentations, including scalp involvement with alopecia or ulceration, may further obscure the diagnosis [6-8]. We report a case illustrating this diagnostic difficulty and highlighting the importance of repeat biopsies in establishing the correct diagnosis.

## Case presentation

**Figure 1:**
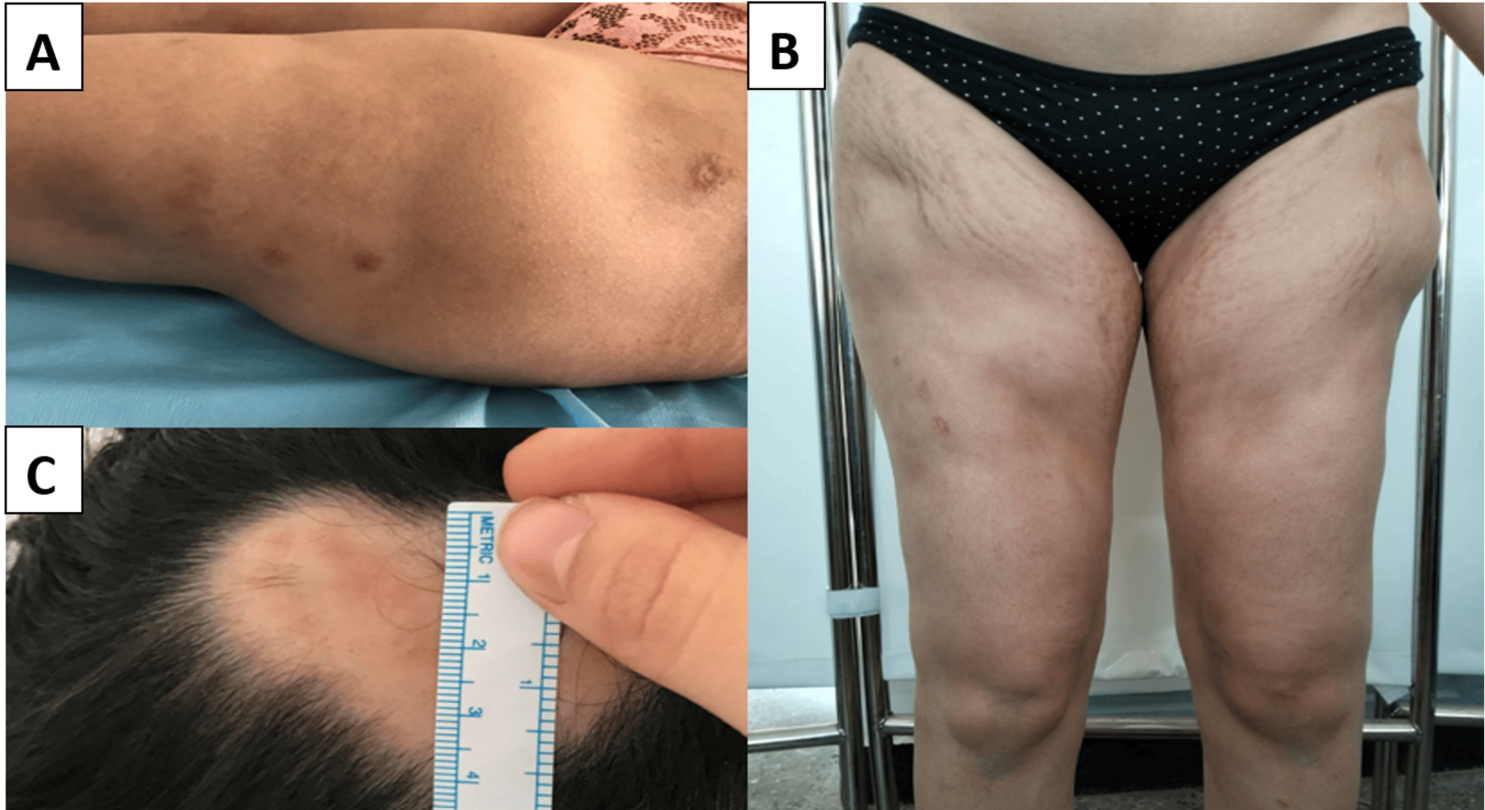
Clinical presentation (A, B) Depressed hyperpigmented plaques with a lipoatrophic appearance involving the thighs. (C) Erythematous alopecic plaque of the scalp at presentation.

A 22-year-old woman with no significant past medical history presented with a three-year history of slowly progressive depressed hyperpigmented plaques with a lipoatrophic appearance involving the thighs, back, and left upper limb, associated with an erythematous alopecic plaque of the scalp measuring 3 × 7 cm (Figure 1).

The lesions were asymptomatic, and her general condition was preserved, and no constitutional symptoms such as fever, weight loss, or night sweats were reported.

An initial biopsy performed on a thigh lesion showed interface dermatitis, dermal mucin deposition, and lobular lymphocytic panniculitis. Direct immunofluorescence revealed deposits of IgG, IgM, and C3, leading to an initial diagnosis of lupus panniculitis. Hydroxychloroquine was initiated, but no clinical improvement was observed.

**Figure 2:**
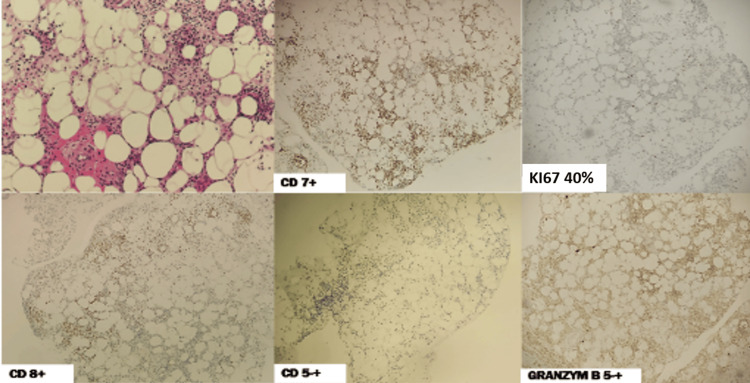
Lobular panniculitis with atypical lymphocytes rimming adipocytes on hematoxylin and eosin staining Immunohistochemistry showed a CD3+, CD8+, CD4-, and CD56- cytotoxic phenotype with granzyme B and perforin expression, with a Ki-67 index of approximately 40%, consistent with subcutaneous panniculitis-like T-cell lymphoma.

Because of persistent progression, a second biopsy of the thigh was performed. Histopathological examination demonstrated lobular panniculitis with atypical lymphoid infiltrate rimming adipocytes. Immunohistochemistry showed CD3+, CD8+, CD4-, and CD56- cytotoxic T lymphocytes expressing granzyme B and perforin, with a Ki-67 proliferation index of 40%, consistent with SPTCL (Figure 2).

Re-evaluation of the initial biopsy with additional immunohistochemical staining retrospectively supported the same diagnosis.

Immunological investigations showed positive antinuclear antibodies and anti-double-stranded DNA antibodies. Laboratory evaluation also showed anemia, leukopenia, and lymphopenia, whereas the platelet count was within the normal range (Table 1).

**Table 1:** Immunological and hematological investigations

Parameter	Result	Reference range	Interpretation
Antinuclear antibodies	0.152778	Negative	Positive
Anti-double-stranded DNA antibodies	28	<10	Increased
Hemoglobin	10.4 g/dL	12-16 g/dL	Decreased
Leukocyte count	3220/mm^3^	4000-10,000/mm^3^	Decreased
Lymphocyte count	1000/mm^3^	1500-4000/mm^3^	Decreased
Platelet count	197,000/mm^3^	150,000-400,000/mm^3^	Normal

18F-FDG PET/CT showed subcutaneous hypermetabolic foci involving the abdominal wall, thighs, and lower back, without lymph node or bone marrow involvement. A bone marrow biopsy and lymph node biopsy were normal. No hemophagocytic syndrome was detected.

The patient was treated with oral corticosteroids at 0.5 mg/kg/day and topical minoxidil for scalp alopecia. After four months, no new lesions were observed, the subcutaneous nodules had completely resolved, and partial hair regrowth was noted on the scalp. One year later, the patient relapsed with the appearance of new nodules, and methotrexate 15 mg weekly was added as adjuvant therapy.

**Figure 3:**
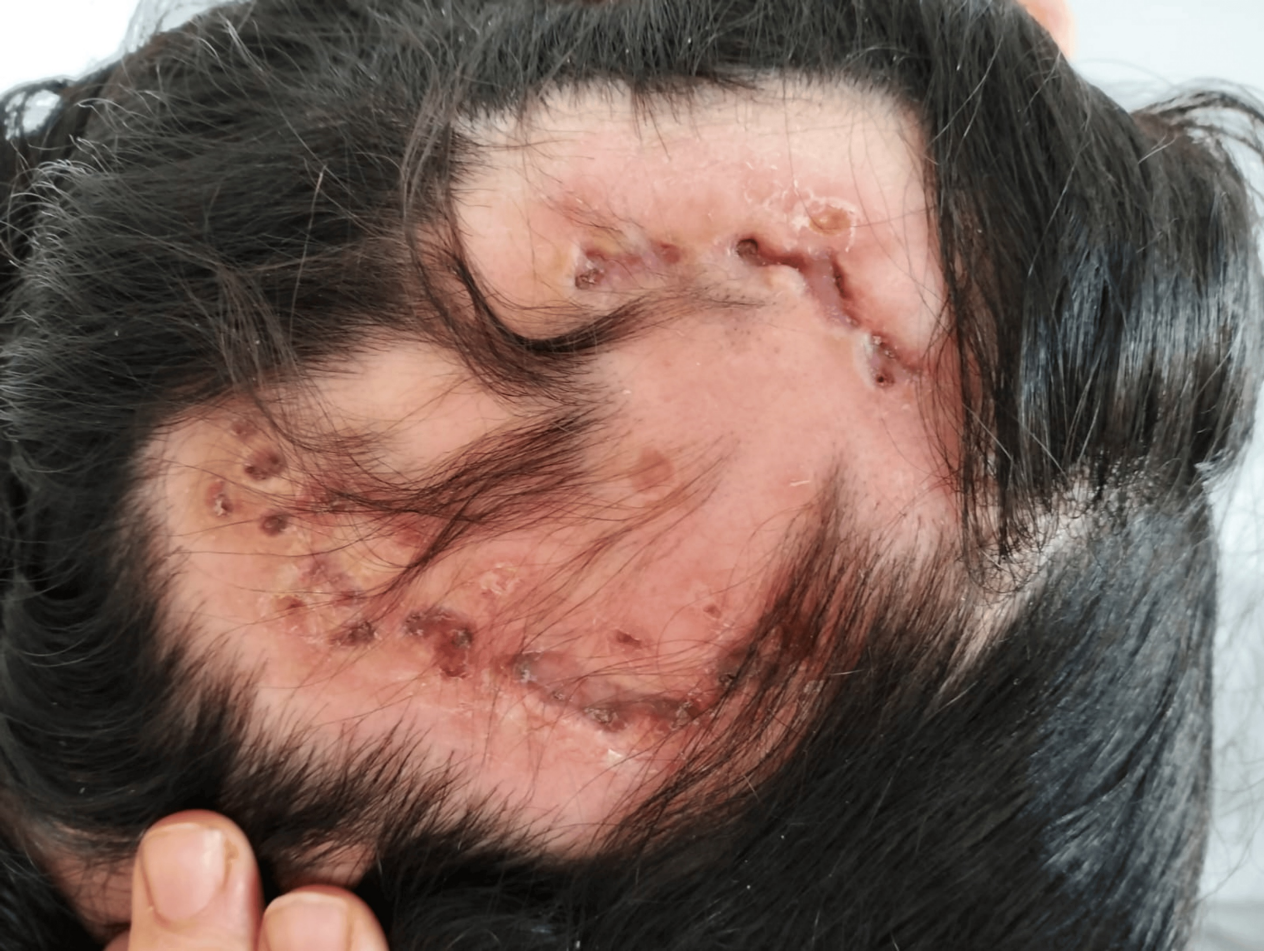
Progressive enlargement of the scalp lesion, reaching 10 cm in greatest diameter, with central vellus hairs and linear ulcerations

After several months of stabilization, the scalp lesion increased in size, reaching 10 cm in greatest diameter, with central vellus hairs and linear ulcerations (Figure 3).

A repeat scalp biopsy with immunohistochemistry showed findings consistent with SPTCL, confirming persistent scalp involvement. The same systemic treatment was continued, and intralesional corticosteroid injections were added. Thereafter, the lesions remained clinically stable without unfavorable systemic progression.

## Discussion

SPTCL is a rare primary cutaneous lymphoma characterized by predominant subcutaneous infiltration of cytotoxic CD8+ T lymphocytes [1-3]. Although it generally carries a more favorable prognosis than primary cutaneous gamma-delta T-cell lymphoma, delayed diagnosis may influence management and clinical outcome [3,9].

Clinically, SPTCL usually presents as deep-seated nodules or plaques involving the trunk and extremities [9,10]. Systemic symptoms may occur but are not constant. In our patient, the disease presented with depressed hyperpigmented plaques exhibiting a lipoatrophic appearance, together with scalp involvement manifesting as an erythematous alopecic plaque. The indolent course and absence of systemic manifestations initially supported a diagnosis of lupus panniculitis.

Scalp involvement in SPTCL appears to be uncommon and may present as alopecia, inflammatory plaques, or ulcerated lesions, thereby mimicking inflammatory or autoimmune disorders [6-8]. In our case, progressive enlargement and ulceration of the scalp lesion prompted repeat biopsy, which confirmed persistent lymphomatous infiltration. Similar diagnostic difficulty has been reported in previous cases in which SPTCL presented clinically with alopecia or other atypical scalp lesions [6,7]. More recent data on primary cutaneous lymphomas involving the scalp also support the diagnostic relevance of this localization [8]. This highlights the importance of reassessing atypical or progressive scalp lesions, particularly when therapeutic response is incomplete or unsatisfactory.

Histologically, SPTCL is characterized by lobular panniculitis with atypical lymphocytes rimming adipocytes and expressing a cytotoxic CD8+, granzyme B+, and perforin+ phenotype [4,9,10]. The absence of CD56 expression in our patient favors alpha-beta SPTCL rather than primary cutaneous gamma-delta T-cell lymphoma, which is associated with a poorer prognosis [3,10]. Another CD56-negative cutaneous T-cell lymphoma that may enter the differential diagnosis is mycosis fungoides. However, unlike SPTCL, mycosis fungoides typically shows epidermotropism with atypical lymphocytes involving the epidermis and superficial dermis, rather than a predominantly lobular panniculitis with adipocyte rimming [11]. At the same time, the initial presence of interface change, dermal mucin deposition, and positive direct immunofluorescence created a significant clinicopathologic overlap with lupus panniculitis and contributed to diagnostic delay.

Autoimmune abnormalities have been reported in approximately 20% of SPTCL cases and may further complicate the diagnostic process [5,12]. In our patient, positive antinuclear and anti-double-stranded DNA antibodies, together with interface dermatitis, dermal mucin deposition, and positive direct immunofluorescence, supported the initial diagnosis of lupus panniculitis and may have contributed to diagnostic delay. These findings may be seen in cases showing clinicopathologic overlap between SPTCL and lupus panniculitis [5,12]. Therefore, autoimmune serology should be interpreted in conjunction with the overall clinicopathologic findings rather than in isolation.

There is no standardized treatment for SPTCL. Immunosuppressive therapy, including systemic corticosteroids and methotrexate, may achieve disease control in selected cases [12]. More aggressive treatment may be required in patients who develop hemophagocytic syndrome [13]. In our case, treatment response was assessed clinically and included absence of new lesions, complete regression of subcutaneous nodules, partial hair regrowth, and subsequent stabilization, although scalp involvement persisted and required additional intralesional corticosteroid injections.

Overall, this case emphasizes the need for early reconsideration of the diagnosis and repeat biopsies, particularly in the presence of progressive or atypical scalp involvement. It also illustrates how SPTCL may show marked overlapping clinicopathologic features with lupus panniculitis, making close clinicopathologic correlation crucial for accurate diagnosis and appropriate management.

## Conclusions

This case highlights the substantial diagnostic overlap between lupus panniculitis and subcutaneous panniculitis-like T-cell lymphoma. Lupus-like clinical, immunological, and histopathological features may initially mask the diagnosis of SPTCL and delay appropriate management. Uncommon scalp involvement, especially when presenting with alopecia and later ulceration, may represent an additional diagnostic pitfall. Repeated biopsy with immunohistochemical reassessment is essential in progressive, atypical, or treatment-refractory panniculitic lesions. Early clinicopathological reconsideration may help avoid misdiagnosis and improve patient management.
